# The Impact on Healthcare Workers of Italian Law n. 24/2017 “Gelli–Bianco” on Patient Safety and Medical Liability: A National Survey

**DOI:** 10.3390/ijerph19148448

**Published:** 2022-07-11

**Authors:** Giuseppe Davide Albano, Arianna Rifiorito, Ginevra Malta, Erika Serena Sorrentino, Vincenzo Falco, Alberto Firenze, Antonina Argo, Stefania Zerbo

**Affiliations:** 1 PROMISE Department, University of Palermo, Piazza Marina, 61, 90133 Palermo, Italy; arianna.rifiorito@unipa.it (A.R.); ginevra.malta@unipa.it (G.M.); erikaserena.sorrentino@community.unipa.it (E.S.S.); alberto.firenze@unipa.it (A.F.); antonella.argo@unipa.it (A.A.); stefania.zerbo@unipa.it (S.Z.); 2 Department of Economics, Business, and Statistics, University of Palermo, Viale delle Scienze, Building 13, 90128 Palermo, Italy; vincenzo.falco01@unipa.it

**Keywords:** law “Gelli–Bianco”, patient safety, medical malpractice, medical liability, clinical risk management, survey, healthcare workers

## Abstract

Italian “Gelli–Bianco” law (law n. 24 enacted by the Italian Government on 8 March 2017) introduced innovative changes and regulations regarding patient safety and healthcare workers’ (HCWs) liability. We promoted a national survey to evaluate the effect of the law on HCWs. The questionnaire was edited and distributed using the free online tool “Google Forms” (Google LLC). The mode of administration chosen for the questionnaire was telematic self-completion. In particular, the questionnaire was sent to several portals of information, websites, in the scientific and medical sectors. Four hundred forty-five subjects participated in the survey. The differences in categorical variables for Gelli–Bianco Law reading with professional variables were analyzed in a univariate analysis using the Chi-square test and Fisher’s exact test. Reading the law is significantly and positively related to knowledge and communication of adverse events and sentinel events, checklist adoption, and participation in educational activities on risk management. The law’s implementation and promotion is a reliable educational tool for increasing patient safety culture and involving HCWs in risk management activities. Knowledge of the law, related education, and understanding of its application are still inadequate; therefore, educational programs regarding patient safety, risk management, and the contents of the law itself must be vigorously promoted to achieve clinical governance goals.

## 1. Introduction

Italian “Gelli–Bianco” law (law n. 24 enacted by the Italian Government on 8 March 2017 [1]) introduced innovative changes and regulations regarding patient safety and healthcare workers’ (HCWs) liability (Table 1). The first article recognized patient safety as a fundamental right and noted that the National Health Service’s primary goal is the prevention of harm to patients in healthcare facilities [2]. It has been over two decades since the report To Err Is Human was published by The Institute of Medicine. This report emphasized the importance of organization and working environment for keeping patients safe, raising concerns about practices that place them at risk. Achieving a safety culture requires an understanding of the values and attitudes that are appropriate and expected for prioritizing patient safety. In this regard, a compliant working environment is crucial for reducing the likelihood of errors and guaranteeing patient care quality and safety. Staffing levels, workload, physical environment, working organization, and communication are associated with quality of care and patient outcomes. The professionalism and interprofessional skills of nurses are essential for clinical safety and the health system as a whole. The work environment is considered a complex system, and greater empowerment of professionalism can result in improved patient safety. In the “Gelli–Bianco” law, there is no distinction between medical doctors and other healthcare workers. Indeed, according to article n. 1, all healthcare workers must participate in risk management strategies and improve patient safety in healthcare facilities. Furthermore, medical malpractice liability and patient safety significantly affect health system sustainability [3,4]. As a matter of fact, adverse events and unsafe care cause a heavy economic burden for health services; the estimated total cost of compensation related to adverse events was roughly €1 billion (£843 m; $1.1 bn) each year in Italy. Moreover, defensive medicine practices lead to additional costs estimated at around €10 bn a year for healthcare services in our country [5,6,7]. In light of this, the new law focused on reducing the number of medical malpractice claims against HCWs by introducing a better regimen of professional liability that emphasizes clinical practice guidelines validated by the Italian Health Institute. Furthermore, the law encourages risk management actions as a relevant part of clinical governance to reduce the risk of patient injury, but also to improve healthcare standards and achieve clinical excellence [8,9,10,11]. The Italian Health System is very heterogeneous among different regions [12]. Moreover, patient safety culture still needs to be implemented in Italy [13]. According to the law, risk management strategies and patient safety issues represent the basic standard of care that must be achieved at the regional health system level. Education and training are relevant tools to reduce the risk of harmful care in all healthcare settings. The implementation of the law is still evolving, as several years are required to study its social, economic, and jurisprudential impact. Therefore, we promoted a national survey to evaluate the effect of the law on HCWs. This paper presents the results of this survey, providing a full report of the HCWs’ perspective on their knowledge of law n. 24 and its impact on patient safety, medical liability litigation, and daily operational practices.

## 2. Materials and Methods

### 2.1. Study Design

There was no need for International Review Board approval, as no patients were involved. We launched a national survey to get national data about the perception of HCWs on the implementation issues of the “Gelli–Bianco” Law.

The survey explores four main content areas:

(1) Knowledge of the Law and its weaknesses, strengths, limitations, and opportunities;

(2) The current impact on clinical risk management (knowledge of sentinel events, adverse events, near misses, use of checklists);

(3) Communication and relationship between HCWs and patients;

(4) Implications for professional liability.

The Health Department of the “Paolo Giaccone” Hospital in Palermo, Italy, approved the survey. The questionnaire was edited and distributed using the free online tool “Google Forms” (Google LLC, Mountain View, California, CA, USA), as was previously carried out in other surveys [12,14,15]. The mode of administration chosen for the questionnaire was telematic self-completion. In particular, the questionnaire was sent to several portals of information, websites, in the scientific and medical sectors, the websites of the local organizations of healthcare professionals such as medical doctors, dentists, nurses, pharmacists, radiology technicians, and obstetricians (Nurse24.it, Segretariato Italiano Giovani Medici SIGM, Doctor33.it, Odontroiatria33.it, Professionetsrm.it, Farmacista33.it, OMCEO Milano, OMCEO Prato, OMCEO Bergamo, OMCEO Brescia, OMCEO Udine, OMCEO Trapani, OMCEO Palermo, OPI Arezzo, OPI Trieste, OPI Cremona, OPI Agrigento, OPI Ancona, OPI Genova, OPI Bergamo, Ordine TSRM Mantova, Ordine TSRM Cuneo, Ordine TSRM Catania, Ordine TSRM e PSTRP Perugia e Terni). The online administration period ran from 1 February to 30 April 2021. All professional healthcare categories and working areas’ involvement, as well as a wide distribution in national territory (northern, center, and southern area), have been obtained.

The questionnaire was preceded by a brief presentation that explained the purpose of the study, the motivation to respond, the mode of response, the privacy policy, and the data processing method. The participants were informed that the questionnaire would be managed in aggregated form to ensure anonymity. All participants could answer the questionnaire only one time with their personal data and their e-mail contact. Informed consent was obtained from all subjects involved in the study. Consent to participate in the research and support for data processing were also requested. The questionnair was based on 25 questions: 22 closed answers plus two open questions with a free text response. A pilot study was not performed prior to the questionnaire distribution. However the questionnaire was revised by the director of our department, Full Professor in Legal Medicine, and first distributed among our department healthcare workers for validation.

### 2.2. Data Analysis

All analyses were performed using R software (R Core Team. 2013, Vienna, Austria). Firstly, we analyzed the sample’s composition through two- and three-way tables of demographic variables. Secondly, differences in categorical variables for Gelli–Bianco Law knowledge/reading with socio-demographic characteristics and professional variables were analyzed in a univariate analysis using the Chi square test and Fisher’s exact test [16]. Finally, we used the logistic regression model to evaluate the effect of anyvariables on Gelli–Bianco Law knowledge/reading [17]. A *p*-value less than 0.05 was considered significant in this analysis.

## 3. Results

The list of questions and answers is reported in Table 2. 445 subjects (299 females and 146 males; mean age 43.38, standard deviation 10.16) participated in the survey. Regardless of gender, most respondents were over the age of 40 (55.5% of the females and 60.9% of males) and resided in the North (73.2% of females and 63.0% of males), mean age 43.38, standard deviation 10.16 (Table 3). Most subjects had a bachelor’s degree (83.2%) and were physicians, nurses, or technicians (70.2%). Most physicians, pharmacists, and dentists had at least a bachelor’s degree, while obstetricians, nurses, technicians, and other professionals had a bachelor’s degree (Table 4). Finally, most subjects were tenured (67.2%) and had seniority for fewer than 21 years (59.1%). More than half of respondents (*n* = 273/445, 61.3%) claimed to have read the Gelli–Bianco Law, while 38.7% (*n* = 172) had never read it.

### Sub-Analysis According to Knowledge of the“Gelli–Bianco” Law

As already mentioned, differences in categorical variables for the reading of the Gelli–Bianco Law with professional variables were analyzed in a univariate analysis using the Chi square test and Fisher’s exact test. Among socio-demographic characteristics, age (*p* = 0.041), geographical macro-region (*p* = 0.007), educational qualification (*p* = 0.007), professional qualification (*p* < 0.001), functional position (*p* = 0.026), and working area (*p* < 0.001) were statistically significantly associated with reading of the Gelli–Bianco Law (Table 5).

Among professional characteristics, participation in clinical risk training events (*p* < 0.001), connection to the portal of the “Istituto Superiore di Sanità” (*p* = 0.003), sentinel events knowledge (*p* < 0.001), sentinel event reporting (*p* < 0.001), the importance of clinical risk management to reduce complaints for professional liability (*p* < 0.001), checklist adoption (*p* < 0.001), healthcare professionals involved in compilation of checklist (*p* < 0.001), the use of checklist to reduce errors in the operating room (*p* < 0.001), near miss knowledge (*p* < 0.001), and the importance of reporting near misses to reduce the occurrence of near misses (*p* < 0.001) were statistically significantly associated with reading of the Gelli–Bianco Law (Table 6).

After carrying out an exploratory analysis, we provided an overview of the sample and analyzed marginal associations between exploratory variables and responses. However, these indications could result from unbalancing variables that may influence reading of the Gelli–Bianco Law. A logistic regression model is estimated to account for this statistical pitfall and the nature of the response variable. Table 7 shows the estimates of the regression coefficients of the model for the reading of the Gelli–Bianco Law, standard errors, and *p*-values.

## 4. Discussion

This study represents the first analysis of the impact of law n.24 on national territory among HCWs. The paper reports the results of a national survey performed to assess knowledge of the law and its application among Italian Healthcare providers in different healthcare facilities. All Italian regions and different healthcare providers were included. According to our data, about half of HCWs have never read the law completely. Reading the law is significantly and positively related to the knowledge and communication of adverse events and sentinel events, checklist adoption, and participation in educational activities on risk management. Therefore, the knowledge of the law could be related to a more developed patient safety culture.

The survey did not focus on specific settings such as hospitals and territorial facilities [18]. However, the working area (ambulatory, surgery, clinical, emergency) has been considered among the socio-demographic characteristics, providing helpful information regarding the sample distribution and adherence to the law according to professional profile. Half of the ambulatory/territorial HCWs had never read the law before the survey; meanwhile, in other areas, that percentage is lower, especially and significantly in the clinical area. The Italian experience indicates that seniority may affect the reading of the law. Indeed, the group with less than five years seniority showed a significantly higher probability of having read the law compared to the 21–30 years seniority group. This point could be due to the relatively greater participation of younger counterparts in training events and their subsequently heightened adherence to innovations introduced in healthcare facilities.

This study supports literature data by confirming the importance of patient safety culture in the prevention of adverse events and risk management [18,19,20]. The law addresses and regulates the safety of care and health risk, and the responsibility of the health care provider of public and private health facilities. Therefore, the novelties introduced by the law tend to fill all the gaps present in previous legislation concerning the risk management of healthcare facilities. Healthcare settings are high-reliability organizations in which safety is a high-priority organizational goal that needs to be promoted by continuous training and support [21]. Moreover, several studies suggested a correlation between patient safety culture and adverse event prevention [18,19,20,21,22].The literature and our study highlight the importance of patient safety culture measurement as a management strategy to improve quality in healthcare. In this regard, the application and diffusion of law n.24 could be a helpful tool to promote patient safety culture.

According to questions #4 and #8, about one-fifth of the sample did not recognize a sentinel event and had never participated in an educational meeting to prevent adverse events. Moreover, the replies to question #12 highlight the most frequent factors of adverse events causality: work overload, distraction, fatigue, staff shortages, and absence of procedures and protocols. Consequently, human factors account for most elements involved in patient safety.

Law n. 24 promotes clinical risk management as a fundamental tool to reduce the risk of harmful health assistance and to evaluate all the preventive measures to ensure high quality and safe assistance [15,23]. Moreover, the law introduces innovation regarding medical liability, focusing on and encouraging healthcare facilities litigation rather than direct action against the HCWs. Thus, the Gelli–Bianco law keeps the liability of health care facilities in the field of contractual law work that is still more favorable to patients, hence constituting an effective tool for funneling claims from malpractice victims towards the facilities, which are in a better position than doctors to meet their compensatory demands. In this regard, all adverse events are a potential source of economic damage and affect the sustainability of the health system [22,24]. Risk management methods and tools are a part of clinical governance, and are aimed at continuously improving healthcare standards and the quality and safety of assistance. The HCWs’ role is crucial in this process to achieve safety goals and adopt adequate preventive measures [25].

Our data demonstrate that half of the HCWs had received inadequate training regarding risk prevention and patient safety. The crucial element of this new regulatory plan is the precise identification of professional profiles that can access the figure of the Risk Manager. From this, an articulated definition of the roles and responsibilities of this new figure can be derived, indicating the absolute need for an adequate and updated training process. Educational programs play a pivotal role in the formulation of risk management strategies and the improvement of patient safety culture. Moreover, training is a helpful tool for preventing and surveilling adverse events, leading to a significant decrease in healthcare facilities’ litigation and costs [15,23,26]. The law highlights the importance of following evidence-based guidelines and good clinical practices in daily activity. According to our data, HCWs who have read law n.24 tend to be better at reporting adverse events and near misses, and maintain a greater patient safety culture. Thus, the diffusion and explanation of the law and its contents may play a role in HCWs’ educational programs to guarantee patient safety.

The World Health Organization estimates the rate of adverse events, based on numerous studies conducted in multiple countries, to be about 10% [27]. This means that ten out of a hundred hospitalized patients suffer damage related to their care rather than the evolution or complication of their disease itself. Nearly half of these adverse events are preventable. The perspective used in the research on adverse events is, in almost all studies, based on the quality of care and not on the search for responsibility. Following the search results in this context, an international movement has been established that has developed theories, methods, and tools to introduce changes in clinical practice to reduce adverse events. An important new element was addressing the safety of all outside the legal and liability aspects that were commonly considered, at least in our country. Article 1 of the law highlights a relatively innovative approach to the safety of care, no longer based solely and exclusively on the skills and knowledge of the individual healthcare provider, but on the organization as a whole [2,28]. Patient safety emerges from the interaction between all the system components, and it does not depend solely on people, technologies, or the organization, but on the interaction between them. This approach is based on the concept that errors and human behavior cannot be understood and analyzed in isolation, rather that the context in which people work must be taken into account. Medical and health personnel are affected by the nature of the tasks they perform, the workgroup, the work environment, and the broader organizational context, that is, the so-called systemic factors. From this perspective, errors can be seen as the consequences of more general problems in the workplace and the organization.

Law n.24 is still being implemented, and several years are needed to assess the effects on patient safety culture and litigation prevention to achieve the stated goals by monitoring adverse events and reducing medical liability and the costs of the healthcare system [29,30]. Several studies suggested that educational programs and patient safety culture improvement lead to a reduction in the number of adverse events and greater patient and HCW safety perspectives in healthcare facilities [31,32,33]. Moreover, the promotion of guidelines and good clinical practices mandated by Law n. 24 leads to a standardization of HCW practices that implement quality assistance and decrease the risk of harmful assistance [24].

Our study has some limitations. The Italian health system is very heterogeneous, and every region can be considered an isolated health system with its own strategies, goals, and management. Patient safety culture and the application of the law are different among different regions and districts. Every district has its own local health unit and public health department, and there are significant differences in patient safety culture between hospitals and territory facilities [6,18,22]. Moreover, the chosen modality was the self-completion of the questionnaire, and the sample is not probabilistic and not wholly representative of the entire population of health professionals. The method of participation was not balanced according to healthcare categories and other demographic factors. The law aims to standardize all regions’ risk management activities by promoting communication and data transmission regarding adverse events, near misses, sentinel events, and malpractice litigation data. Further efforts are needed to achieve this goal. Implementation and promotion of the law may help to improve patient safety culture and harmonize risk management strategies in the national territory. Other countries should be aware of the importance of introducing regulations regarding patient safety and medical liability when aiming to improve their healthcare systems.

## 5. Conclusions

The survey offers a snapshot of the current impact of Italian law n.24 on patient safety and medical liability in the national territory. The implementation and promotion of the law could be a reliable educational tool for increasing patient safety culture and involving HCWs in risk management activities. Knowledge of the law, education, and application are still inadequate, and half of the HCWs who participated in the survey received inadequate training regarding risk prevention and patient safety; therefore, educational programs regarding patient safety, risk management, and law contents must be vigorously promoted to achieve clinical governance goals. The government and the national and local health institutions should improve their efforts to harmonize adherence to the law in the national territory and encourage standardized risk management strategies. Additional research should include a homogenous sample and healthcare setting, and could focus on the effects of the law’s impact after an educational and patient safety program.

## Figures and Tables

**Table 1:** A summary of the key-points of “Gelli–Bianco” law about patient safety and medical liability.

Key Points of Law n. 24/2017 “Provisions on Safety of the Healthcare and of the Patient, as Well as on Professional Liability of the Healthcare Providers”
**Article 1:** “the safety of healthcare is an essential part of the constitutional right of health and it is pursued on behalf of the individual and of the community”; **Article 1:** all healthcare workers must participate in risk management strategies and improving patient safety in healthcare facilities. **Article 3:** establishes a National observatory for good clinical practices and patient safety aimed at collecting all national data in health care facilities concerning risks and adverse events as well as promoting patient safety culture and evidence-based guidelines; **Article 5:** highlights the importance of following the recommendations provided by guidelines drawn up by qualified selected scientific institutions and published on the National Health System website; **Article 6:** specifically deals with the criminal liability of healthcare providers, introducing a new article in the Italian Criminal Code: art. 590-sexies. HCWs’ practice is evaluated according to adherence to guidelines and good clinical practices recommendations; **Article 7:** deals with malpractice litigation by distinguishing the HCW’s liability from the liabilities of the Healthcare facility itself. Healthcare facility litigation is encouraged rather than direct action against HCWs. HCW’s practice is evaluated according to adherence to guidelines and good clinical practices recommendations.

**Table 2:** Full list of questions and answers obtained from the survey on Gelli–Bianco Law.

Question	Total Answer	Answer	
1. Have you read the contents of the Gelli–Bianco Law?	445/445 (100%)	No 172 (38.7%) Yes 252 (56.6%) Partly 21 (4.7%)	
2. If so, do you believe the law has made proper corrections to previous regulations? In what terms?	281/445 (63.1%)	-Greater security for the counterparts of consultants and judges in assessing professional liability 138 (49.15%) -Greater patient safety 103 (36.7%) -Greater protection for HCWs 135 (48%) -Positive effects on reshaping insurance policy costs 45 (16%)	
3. Do you think the Gelli–Bianco Law has any limitations?	418/445 (93.9%)	-No 94 (21.1%) -Yes 143 (32.1%) -Does not know 181 (40.6%)	
4. Have you ever participated in clinical risk training events? If so, what kind?	445/445 (100%)	No 81 (18.2%) Yes 364 (81.8%)	-master’s topic 57 (15.6%) -specific master 18 (4.9%) -online course 93 (25.5%) -subject of the degree course 85 (23.3%) -in hospital course 164 (45%) -out of hospital course 82 (18.4%)
5. Do you feel you have received adequate training regarding risk prevention and patient safety?	445/445 (100%)	No 222 (49.9%) Yes 223 (50.1%)	
6. Do you feel that risk management should involve: all operators; only those working in the operating room; all physicians; only nurses?	445/445 (100%)	Only nurses 1 (0.2%) All operators 444 (99.8%)	
7. Have you ever connected to the website of the Istituto Superiore di Sanità https://www.iss.it/linee-guida1?	445/445 (100%)	No 183 (41.1%) Yes 262 (58.9%)	
8. Do you know what sentinel events are?	445/445 (100%)	No 77 (17.3%) Yes 368 (82.7%)	
9. In the course of your work, have sentinel events ever occurred, and if so, which ones?	445/445 (100%)	No 233 (52.4%) Does not know 66 (14.8%) Yes 56 (12.6%)	-suicide or attempted suicide of patient (reported 12 times) -act of violence against operator (reported 11 times) -procedure in wrong patient (reported 5 times) -transfusion reaction due to AB0 incompatibility (reported 3 times)
10. Have you ever reported a sentinel event and if so, to whom did you report it?	445/445 (100%)	No 298 (67%) Yes 147 (33%)	-Unit Director 39 (8.8%) -Nursing of technical coordinator 54 (12.1%) -Health director 39 (8.8%) -Risk manager 60 (13.5%) -General manager 9 (2%)
11. Do you think that clinical risk management can contribute to reduce complaints for professional liability?	445/445 (100%)	No 22 (4.9%) Yes 369 (82.9%) Does not know 54 (12.1%)	
12. During the care provided to the patient, have you ever committed errors that did not cause harm to the patient and, if so, why do you think the error occurred?	445/445 (100%)	No 131 (29.4%) Yes 314 (70.6%)	-Work overload 179 (40.2%) -Distraction 115 (25.8%) -Fatigue 109 (24.5%) -Staff shortages 100 (22.5%) -Absence of procedures 79 (17.8%) -Forgetfulness of procedures 34 (7.6%)
13. Have you ever received a warranty notice?	445/445 (100%)	No 22 (4.9%) Yes 423 (95.1%)	
14. During your professional activity have you been involved in judicial investigations and if so, at what stage is the judicial process?	445/445 (100%)	-No 393 (87.9%) -Yes 52 (12.1%)	-Proceedings are currently without follow-up 24 (5.4%) -Preliminary stage of the investigation 14 (3.1%) -Definitively acquitted 10 (2.2) -Second degree of judgment 2 (0.4%) -Definitely condemned 1 (0.2%) -Closure 1 (0.2%)
15. Do you also carry out your work as a self-employed?	445/445 (100%)	No 307 (69%) Yes 138 (31%)	
16. Have you ever received threats or aggression from patients or their families?”	445/445 (100%)	No 287 (64.5%) Yes 158 (35.5%)	
17. Have you sustained any injuries for which you had to undergo therapy?	445/445 (100%)	No 412 (92.6%) Yes 33 (7.4%)	
18. Have you ever been involved in a work-related injury?	445/445 (100%)	No 311 (69.9%) Yes 134 (30.1%)	
19. Which risk management activity would you like to see implemented by the healthcare facility you work in?	401/445 (90.1%)	-Does not know 114 (25.6%) -Periodic company training and refresher courses 104 (23.3%) -Clinical audits 18 (4%) -RCA (Root cause analysis) 5 (1.12%) -FMECA (Failure Mode, Effects, and Criticality Analysis) 6 (1.3%) -Greater promotion of the incident report system 29 (6.5%) -Event simulation 7 (1.5%) -Promotion of the use of clear and shared protocols 35 (7.8%) -Burnout risk 9 (2%) -Adequate distribution of workloads 44 (9.8%) -Training on professional patient communication 20 (4.4%) -Review of procedures adopted 10 (22.4%)	
20. In your unit have the checklists promoted by the Ministry of Health been adopted?	445/445 (100%)	No 107 (24%) Yes 169 (38%) Does not know 169 (38%)	
21. What type HCWs are involved in their compilation?	445/445 (100%)	Does not know 252 (56.6%) Only anesthesiologist 3 (0.7%) Only nurse 35 (7.9%) Only surgeons 3 (0.7%) All operators 152 (34.2%)	
22. Do you think the use of the checklist will help to reduce errors in the operating room?	445/445 (100%)	No 14 (3.1%) Yes 324 (72.8%) Does not know 107 (24%)	
23. Do you believe that the proper completion of medical records and all health care documentation can help reduce professional liability claims	445/445 (100%)	No 20 (4.5%) Yes 404 (90.8%) Does not know 21 (4.7%)	
24. Have you ever heard of near misses?	445/445 (100%)	No 185 (41.6%) Yes 260 (58.4%)	
25. Is voluntary reporting of near misses helpful in reducing the occurrence of near misses?	445/445 (100%)	No 11 (2.5%) Yes 344 (77.3%) Does not know 90 (20.2%)	

**Table 3:** Distribution of subjects by gender, age, and geographical macro-region.

Females												
	18–30		31–40		41–50		51–60		>60		Total	
	*n*	%	*n*	%	*n*	%	*n*	%	*n*	%	*n*	%
North	41	18.7	53	24.2	67	30.6	38	17.4	20	9.1	219	73.2
Center	8	20.0	7	17.5	16	40.0	7	17.5	2	5.0	40	13.4
South	12	30.0	12	30.0	6	15.0	5	12.5	5	12.5	40	13.4
Total	61	20.4	72	24.1	89	29.8	50	16.7	27	9.0	299	
Males												
North	17	18.5	23	25.0	16	17.4	19	20.7	17	18.5	92	63.0
Center	2	15.4	3	23.1	1	7.7	5	38.5	2	15.4	13	8.9
South	4	9.8	8	19.5	8	19.5	13	31.7	8	19.5	41	28.1
Total	23	15.8	34	23.3	25	17.1	37	25.3	27	18.5	146	

**Table 4:** Distribution of subjects by educational and professional qualification.

	High School		University Degree		Graduation		Master Degree		Total	
	*n*	%	*n*	%	*n*	%	*n*	%	*n*	%
Medical Doctor	0	0.0	1	0.9	28	25.7	80	73.4	109	24.5
Pharmacist	0	0.0	0	0.0	1	11.1	8	88.9	9	2.0
Obstetrician	0	0.0	3	20.0	11	73.3	1	6.7	15	3.4
Dentist	0	0.0	0	0.0	3	30.0	7	70.0	10	2.2
Nurse	9	10.3	14	16.1	39	44.8	25	28.7	87	19.6
Technician	3	2.6	18	15.5	67	57.8	28	24.1	116	26.1
Other	1	1.0	26	26.3	57	57.6	15	15.2	99	22.2
Total	13	2.9	62	13.9	206	46.3	164	36.9	445	

**Table 5:** Socio-demographic characteristics of subjects of the study sample and association in the univariate analysis to Gelli–Bianco Law reading (*n = 428*).

		Gelli–Bianco Law Reading				*p*-Value
		No (*n* = *172*)		Yes (*n* = *273*)		
		*n*	%	*n*	%	
Gender	F	119	39.8	180	60.2	0.543
	M	53	36.3	93	63.7	
Age	18–30	40	47.6	44	52.4	0.041
	31–40	48	45.3	58	54.7	
	41–50	40	35.1	74	64.9	
	51–60	24	27.6	63	72.4	
	>60	20	37.0	34	63.0	
Geographicaldistribution	North	135	43.4	176	56.6	0.007
	Center	15	28.3	38	71.7	
	South	22	27.2	59	72.8	
Educational qualification	Diploma	3	23.1	10	76.9	0.007
	Universitydegree	24	38.7	38	61.3	
	Graduation	96	46.6	110	53.4	
	Master degree	49	29.9	115	70.1	
Professional qualification	Medical Doctor	44	40.4	65	59.6	<0.001
	Pharmacist	1	11.1	8	88.9	
	Obstetrician	7	46.7	8	53.3	
	Dentist	2	20.0	8	80.0	
	Nurse	13	14.9	74	85.1	
	Technician	52	44.8	64	55.2	
	Other	53	53.5	46	46.5	
Functional position	Fixed-termcontract	21	47.7	23	52.3	0.026
	Permanentcontract	101	33.8	198	66.2	
	Self-employed	40	50.0	40	50.0	
	Other	10	45.5	12	54.5	
Seniority	<5 years	46	42.2	63	57.8	0.031
	5—10 years	32	51.6	30	48.4	
	11—20 years	38	41.3	54	58.7	
	21–30 years	27	281	69	71.9	
	>30 years	29	33.7	57	66.3	
Work area	Medical services	78	51.7	73	48.3	<0.001
	Surgery	13	25.5	38	74.5	
	Emergency	11	22.4	38	77.6	
	Clinical area	70	36.1	124	63.9	

**Table 6:** Questionnaire answers of subjects of the study sample and association in the univariate analysis to their reading of the Gelli–Bianco Law (*n* = 428) (*n*, row%).

		Gelli–Bianco Law Reading				*p*-Value
		No (*n* = 172)		Yes (*n* = 273)		
		*n*	%	*n*	%	
Have you ever participated in clinical risk training events?	No	53	63.1	31	36.9	<0.001
	Yes	119	33.0	242	67.0	
Do you feel you have received adequate corporate training regarding risk prevention and patient safety?	No	93	41.9	129	58.1	0.192
	Yes	79	35.4	144	64.6	
Do you feel that risk management should involve?	Only nurses	0	0.0	1	100.0	0.999
	All operators	172	38.7	272	61.3	
Have you ever connectedto the website of“IstititutoSuperiore di sanità”?	No	86	47.0	97	53.0	0.003
	Yes	86	32.8	176	67.2	
Do you know what sentinel events are?	No	54	70.1	23	29.9	<0.001
	Yes	118	32.1	250	67.9	
Have you ever reported a sentinel event?	No	136	46.1	159	53.9	<0.001
	Yes	36	24.0	114	76.0	
Do you think that clinical risk management can contribute to reduce complaints for professional liability?	No	6	27.3	16	72.7	<0.001
	Does not know	28	51.9	26	48.1	
	Yes	138	37.4	231	62.6	
During the care provided to the patient, have you ever committed errors that did not cause harm to the patient?	No	57	41.6	80	58.4	0.454
	Yes	115	37.3	193	62.7	
Have you ever received a warranty notice?	No	163	38.5	260	61.5	0.999
	Yes	9	40.9	13	59.1	
During your professional activity have you been involved in judicial investigations?	No	155	39.6	236	60.4	0.315
	Yes	17	31.5	37	68.5	
Have you ever received threats or aggression from patients or their families?	No	117	40.8	170	59.2	0.257
	Yes	55	34.8	103	65.2	
Have you sustained any injuries for which you had to undergo therapy?	No	161	39.1	251	60.9	0.641
	Yes	11	33.3	22	66.7	
Have you ever been involved in a work-related injury?	No	127	40.8	184	59.2	0.182
	Yes	45	33.6	89	66.4	
In the O.U. in which you work, have the check list promoted by the Ministry of Health been adopted?	No	34	31.8	73	68.2	<0.001
	Does not know	87	51.5	82	48.5	
	Yes	51	30.2	118	69.8	
Which healthcare professionals are involved intheir compilation?	Does not know	122	48.4	130	51.6	<0.001
	All anesthesiologist	0	0.0	3	100.0	
	All nurses	7	20.0	28	80.0	
	All surgeons	2	66.7	1	33.3	
	All operators	41	27.0	111	73.0	
Do you think the use of the checklist will help reduce errors in the operating room?	No	5	35.7	9	64.3	<0.001
	Does not know	61	57.0	46	43.0	
	Yes	106	32.7	218	67.3	
Do you believe that the completion of all health care documentation can help reduce professional lability?	No	5	25.0	15	75.0	0.414
	Does not know	9	42.9	12	57.1	
	Yes	158	39.1	246	60.9	
Have you ever heard of nearmisses?	No	105	56.8	80	43.2	<0.001
	Yes	67	25.8	193	74.2	
Is voluntary reporting of near misses helpful in reducing the occurrence of near misses?	No	3	27.3	8	72.7	<0.001
	Does not know	53	58.9	37	41.1	
	Yes	116	33.7	228	66.3	

**Table 7:** Results of the logistic regression model on the Bianco-Gelli Law reading.

Coefficients		Estimates	Standard Errors	*p*-Value
Intercept		−1.940	0.424	<0.001
Near miss knowledge	Yes	0.940	0.250	<0.001
Professional qualification	Pharmacist	1.018	1.111	0.359
(baseline: medical doctor)	Obstetrician	−0.914	0.601	0.129
	Dentist	1.902	0.893	0.033
	Nurse	0.734	0.398	0.065
	Technician	−0.189	0.312	0.546
	Other	−0.191	0.331	0.564
Sentinel event knowledge	Yes	0.791	0.332	0.017
Healthcare professionals involved in compilation of checklist	Only anesthesiologists	16.270	715.501	0.982
(baseline: does not know)	Only nurses	0.803	0.485	0.098
	Only surgeons	−0.902	1.405	0.521
	All operators	0.614	0.257	0.017
Participation in clinical risk training events	Yes	0.882	0.304	0.004
Sentinel Eventreporting	Yes	0.552	0.262	0.035
Seniority	5–10 years	0.138	0.258	0.594
(baseline: <5 years)	11–20 years	0.268	0.253	0.290
	21–30 years	−0.690	0.281	0.014
	>30 years	−0.182	0.269	0.498
Work area	Surgery	0.1595	0.4633	0.731
(baseline: Medical Services)	Emergency	0.7349	0.433	0.090
	Clinical area	0.1576	0.2865	0.582

## Data Availability

All data are included in the main text.
